# An Active 32-kDa Cathepsin L Is Secreted Directly from HT 1080 Fibrosarcoma Cells and Not via Lysosomal Exocytosis

**DOI:** 10.1371/journal.pone.0145067

**Published:** 2015-12-16

**Authors:** Yoko Hashimoto, Chihiro Kondo, Nobuhiko Katunuma

**Affiliations:** 1 Department of Biochemistry, School of Dentistry, Aichi-Gakuin University, Nagoya, Japan; 2 Institute for Health Sciences, Tokushima Bunri University, Tokushima, Japan; Institute of Molecular and Cell Biology, Biopolis, UNITED STATES

## Abstract

Cathepsin L [EC 3.4.22.15] is secreted via lysosomal exocytosis by several types of cancer cells, including prostate and breast cancer cells. We previously reported that human cultured fibrosarcoma (HT 1080) cells secrete cathepsin L into the medium; this secreted cathepsin is 10-times more active than intracellular cathepsin. This increased activity was attributed to the presence of a 32-kDa cathepsin L in the medium. The aim of this study was to examine how this active 32-kDa cathepsin L is secreted into the medium. To this end, we compared the secreted active 32-kDa cathepsin L with lysosomal cathepsin L by using a novel gelatin zymography technique that employs leupeptin. We also examined the glycosylation and phosphorylation status of the proteins by using the enzymes endoglycosidase H [EC 3.2.1.96] and alkaline phosphatase [EC 3.1.3.1]. Strong active bands corresponding to the 32-kDa and 34-kDa cathepsin L forms were detected in the medium and lysosomes, respectively. The cell extract exhibited strong active bands for both forms. Moreover, both forms were adsorbed onto a concanavalin A-agarose column. The core protein domain of both forms had the same molecular mass of 30 kDa. The 32-kDa cathepsin L was phosphorylated, while the 34-kDa lysosomal form was dephosphorylated, perhaps because of the lysosomal marker enzyme, acid phosphatase. These results suggest that the active 32-kDa form does not enter the lysosomes. In conclusion, our results indicate that the active 32-kDa cathepsin L is secreted directly from the HT 1080 cells and not via lysosomal exocytosis.

## Introduction

Lysosomes are cellular organelles that play crucial roles in intracellular protein degradation and recycling [1]. Lysosomes also function in a secretory pathway known as lysosomal exocytosis, which involves two sequential steps. In the first step, lysosomes are translocated to the vicinity of the cell surface; this process occurs independent of the intracellular calcium [Ca^2+^] concentration [2]. In the second step, the pool of predocked lysosomes fuses with the plasma membrane; this process depends on an increase in the intracellular [Ca^2+^] concentration [2–9]. Increased [Ca^2+^] triggers the fusion of the lysosomes with the plasma membrane. This lysosomal docking and fusion are regulated by a transcription factor EB (TFEB) for lysosome biogenesis and autophagy [8,9]. The fusion of the lysosomes with the plasma membrane is called lysosomal exocytosis. Lysosomal exocytosis is an important process for normal cellular clearance and daily maintenance of cellular homeostasis [1,7]. Lysosomal exocytosis also leads to the extracellular release of lysosomal enzymes [8,9].

Lysosomes contain many kinds of acid hydrolases, including cathepsins. Cathepsin B [EC 3.4.22.1] and L [EC 3.4.22.15] are cysteine proteinases belonging to the papain family that cleave the extracellular matrix when secreted outside the cells. Several independent studies have reported the importance of cathepsins in cancer invasion and metastasis *in vivo*. Recently, lysosomal exocytosis of cathepsins L was demonstrated in cancer [10]; increased cathepsin L secretion paralleled enhanced tumor cell migration and invasion. Inhibition of the gene expression of lysosomal cathepsin B in a glioblastoma cell line and of cathepsin D [EC 3.4.23.5] exocytosis from glioma cells attenuated their migration and invasion [11,12]. Cancer cells can enhance and take over this housekeeping lysosomal secretion and use it to change the extracellular microenvironment to support invasion and metastasis. Previous studies have suggested that cancer cells secrete cathepsins via lysosomal exocytosis [10,12]; however, the underlying molecular mechanism is unknown.

Cathepsin L is a lysosomal enzyme that is synthesized as a preproform [13] and processed into a 41-kDa proform in the Golgi apparatus. The proform has two fates: to be targeted to lysosomes and to be secreted out of the cells. Cathepsin L contains covalently *N*-linked oligosaccharides; the mannose moiety of cathepsin L is phosphorylated by UDP-*N*-acetylglucosamine:lysosomal enzyme *N*-acetylglucosaminylphosphotransferase [EC 2.7.8.17] in the *cis* Golgi [14]. These mannose-6-phosphate (M-6-P) groups are recognized by an M-6-P receptor protein in the *trans* Golgi network (TGN), and then delivered to lysosomes (via endosomes) [15]. The cathepsins dissociate from the receptors at low lysosomal pH, and the phosphate group is removed from the M-6-P moiety by a lysosomal acid phosphatase [8]. Cathepsin L is secreted via lysosomal exocytosis; its secretion is enhanced during tumor cell metastasis [10]. However, the actual mechanism underlying the lysosomal exocytosis of cathepsins is poorly understood.

Cathepsin L is the most unstable lysosomal cysteine proteinase at neutral and alkaline pH [16]. We previously reported that leupeptin, a tight-binding (but reversible) inhibitor of cysteine proteinases, complexes with cathepsin L and protects it from irreversible denaturation in alkaline solutions such as the electrode buffer (pH 8.3) used for sodium dodecyl sulfate-polyacrylamide gel electrophoresis (SDS-PAGE) [17]. The aldehyde group of leupeptin forms a thioester bond with the -SH group of cysteine in the active site of cathepsin L [18] and renders it stable even in alkaline solutions. This stable three-dimensional structure is maintained during SDS-PAGE, thereby preventing the irreversible denaturation of cathepsin L in alkaline solutions. In addition, leupeptin is easily removed from enzymes upon washing of the gel after electrophoresis. On the basis of these results, we developed an improved gelatin zymography technique that employs leupeptin to detect even small amounts of mature cathepsin L on account of their gelatinolytic activities in our previous study [17]. Moreover, this improved zymography technique can clarify both the molecular weights and activities of cathepsin L intermediates, including the pro and mature forms, simultaneously.

In the same study, we also reported that HT 1080 cells secrete cathepsin L into the medium; the activity of the secreted protein is approximately 10-fold higher than that of the intracellular form [17]. The activity was estimated by reducing the incubation time to 2 min to avoid procathepsin L activation [17,19]. The high cathepsin L activity in the medium was attributed to 32-kDa cathepsin L. This active 32-kDa form was not derived from the extracellular processing of 41-kDa procathepsin L by surface activators, as evidenced by the fact that neither form was affected when the cells were cultured in the presence of various proteinase inhibitors [17].

In the present study, we sought to investigate the mechanism underlying the secretion of the active 32-kDa cathepsin L form into the medium by HT 1080 cells. The secretion mechanism was examined by comparing the secreted and lysosomal cathepsin L forms using the improved gelatin zymography method and by employing two enzymes, endoglycosidase H [EC 3.2.1.96] (mannosyl-glycoprotein endo-β-*N*-acetylglucosaminidase H [End H]) and alkaline phosphatase [EC 3.1.3.1] (ALP). Our results provide insights into the molecular mechanism underlying the membrane trafficking of the active 32-kDa cathepsin L form secreted by HT 1080 cells.

## Materials and Methods

### Materials

Leupeptin and Z-Phe-Arg-MCA were purchased from the Peptide Institute (Osaka, Japan). Dulbecco’s modified Eagle’s medium (DMEM) and fetal calf serum (FCS) were purchased from Invitrogen Corp. (Carlsbad, CA). The human fibrosarcoma cell line HT 1080 was purchased from Dainippon Pharmaceutical Co., Ltd. (Osaka, Japan). End H from *Streptomyces griseus* was purchased from Seikagaku Biobusiness Co. (Tokyo, Japan). ALP (P 4245) was purchased from Sigma-Aldrich Co. (St. Louis, MO). Prestained SDS-PAGE standard (broad range) was purchased from Bio-Rad Laboratories (Hercules, CA). Lectin-agarose minicolumn Kit I, which was packed with concanavalin A (lectin from jack bean, Con A), *Lens culinaris* agglutinin (lectin from lentil seed, LCA), *Ricinus communis* agglutinin 120 (lectin from castor bean, RCA120), and wheat germ agglutinin (lectin from wheat germ, WGA), was purchased from Seikagaku Biobusiness Co. (Tokyo, Japan). CA-074 and CLIK-148 were synthesized by Katunuma’s group [20]. All the other chemicals were of analytical grade.

### Cell culture

HT 1080 cells (1 × 10^5^ cells) were seeded in Falcon™ culture dishes (Becton Dickinson & Co., Franklin Lakes, NJ) and cultured in DMEM in the presence of 200 U/mL penicillin, 0.4 mg/mL streptomycin, 0.2 mg/mL kanamycin, 2 μL/mL Fungizone, and 10% FCS. The cells were then passaged at confluence (up to the fifth generation) at 37°C under 5% CO_2_ in air. After reaching confluence, the cells were washed with serum-free DMEM, and then cultured in the same medium for 24 h. The culture medium was removed, and the cells were washed twice with 1 mL of phosphate-buffered saline (PBS) and collected by centrifugation at 700 ×*g* for 10 min. Enzymes were extracted from the cells by homogenization in 20 mM Tris-HCl buffer, pH 6.8. After centrifugation at 11,400 ×*g* for 10 min at 4°C, the resultant supernatant (cell fraction) and culture medium were stored at −80°C.

### Preparation of the lysosomal fraction from HT 1080 cells

HT 1080 cells, which were prepared as described above, were homogenized in 0.25 M sucrose, and the mitochondrial fraction was removed by centrifugation at 3,300 ×*g* for 10 min at 4°C. Then, the lysosomes were precipitated by centrifugation at 25,000 ×*g* for 10 min at 4°C [21,22]. Lysosomal cathepsin L was solubilized with 0.1 M Tris-HCl buffer (pH 6.8); the insoluble fraction was removed by centrifugation at 8,000 ×*g* for 10 min at 4°C.

### Determination of enzyme activities in the HT 1080 cell and medium fractions

Cathepsin L activity was measured by incubating the cell fraction or culture medium with Z-Phe-Arg-MCA as the substrate and inhibitors of cathepsin B (1 μM CA-074) and/or cathepsin L (5 μM CLIK-148) in 50 mM sodium acetate buffer (pH 5.5) for 10 min at 37°C. The amount of 7-amino-4-methylcoumarin liberated from the substrate was estimated fluorometrically in a Shimadzu RF-540 fluorescence spectrometer (Shimadzu Co., Ltd., Kyoto, Japan) at excitation and emission wavelengths of 370 nm and 460 nm, respectively. The net activity was calculated by subtracting the activity in the presence of inhibitors of cathepsin L and cathepsin B from that in the presence of cathepsin B, as described by Inubushi et al. [19]. One unit of enzyme activity was defined as the amount of enzyme required to degrade 1 nmol of substrate per min.

### Gelatin zymography of the HT 1080 cell fraction, lysosomal fraction, and culture medium

The gelatin-degrading activities were examined on 11.5% polyacrylamide gels containing 0.8 mg/mL gelatin, using a slab gel measuring 85 × 80 × 1 mm, as described previously [17,23]. The cell fraction, lysosomal fraction, or culture medium was mixed with 0.1 M Tris-HCl buffer (pH 6.8−7.2) containing 0.1 mM leupeptin. The samples were then mixed with Laemmli’s sample buffer solution (pH 6.8) containing 2% SDS without reducing reagent [17]. Aliquots of the sample solutions were applied onto the gel without heat treatment. SDS-PAGE was performed at a constant current of 25 mA for 80 min in 25 mM Tris-19.2 mM glycine buffer (pH 8.3) containing 0.1% SDS at room temperature; the temperature of the electrode buffer was maintained at 2°C during the electrohporesis. After electrophoresis, the gel was washed with 2.5% Triton X-100 (Sigma-Aldrich Co.) for 1 h at room temperature to remove both SDS and leupeptin, and then incubated in 0.1 M sodium acetate buffer (pH 4.8) containing 20 mM cysteine and 1 mM EDTA for 20 h at 37°C to allow gelatinolytic activity. The gels were then stained with 0.25% Coomassie™ Birilliant Blue R (Sigma-Aldrich Co.) in 50% methanol-10% acetic acid for 1 h and destained in 20% methanol-10% acetic acid. Enzyme activities were detected as negatively stained bands.

### Determination of the carbohydrate species bound to the cathepsin forms

Carbohydrate species attached to the secreted and lysosomal cathepsin L forms were examined using a Lectin-agarose minicolumn Kit I packed with Con A, LCA, RCA120, and WGA, according to the manufacturer’s instructions. Briefly, 400 μL of sample, which was prepared by diluting both cathepsin forms with PBS containing 0.05 mM leupeptin (column buffer), was loaded onto each lectin-packed minicolumn pre-equilibrated with column buffer. The column was then washed with 2 mL of column buffer (this eluate was pooled as the non-adsorption fraction), and the adsorbed materials were eluted from the column with 10 mM methyl-α-D-glucoside for the Con-A agarose column (this eluate was pooled as the adsorption fraction). Both forms were concentrated by using Centricon-10 (Merck Millipore Co., Darmstadt, Germany), and gelatin zymography was performed to examine which fraction contained the 32- and/or 34-kDa cathepsin L.

### Examination of the core proteins of the secreted and lysosomal cathepsin L forms by treatment with endoglycosidase H (End H)

The carbohydrate moiety was removed from the secreted 32-kDa and lysosomal cathepsin L forms by End H treatment as follows: End H (0.1 unit/vial) was solubilized with 100 μL of 10 mM Tris-HCl buffer (pH 7.2) containing 0.1% bovine serum albumin (BSA, Sigma-Aldrich Co.). An aliquot of the cell fraction, lysosomal fraction, or culture medium was mixed with or without 10 mU of End H in 0.1 M sodium acetate buffer (pH 5.0) containing 0.1 mM leupeptin, and then incubated for 30 min at 37°C. Gelatin zymography was performed for each incubation mixture.

### Investigation of the phosphorylation status of the secreted and lysosomal cathepsin L forms by treatment with alkaline phosphatase (ALP)

An aliquot of the cell fraction, lysosomal fraction, or culture medium was mixed with or without ALP in 0.5 mM Tris-HCl buffer (pH 9.3 at 25°C) containing 0.1 mM leupeptin, 0.1 mM MgCl_2_, 10 μM ZnCl_2_, and 0.1 mM spermidine, and incubated for 30 min at 37°C. Gelatin zymography was performed for each incubation mixture.

### Examination of the processing forms of the secreted and lysosomal cathepsin L forms

A portion of the lysosomal fraction or culture medium was incubated in 0.1 M sodium acetate buffer (pH 4.8) for 0, 2, or 4 h at 37°C in the presence or absence of leupeptin. After incubation, aliquots of the incubation mixtures were subjected to gelatin zymography to examine whether or not the secreted and lysosomal cathepsin L forms were processed further.

## Results

### Detection of lysosomal and secreted cathepsin L by the novel improved gelatin zymography technique

**Fig 1:**
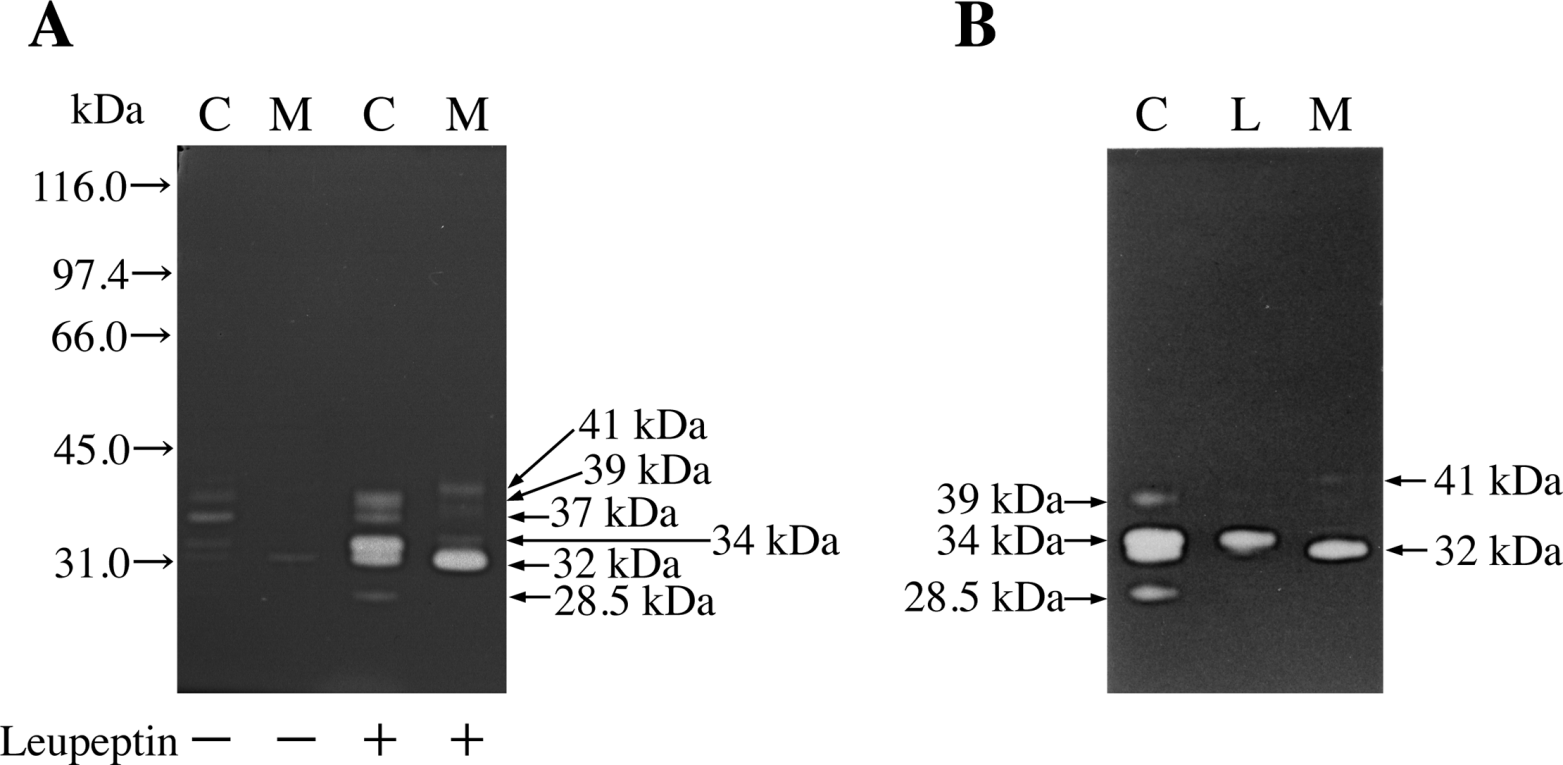
Zymogram of gelatinolytic activities. (A) Zymogram of the gelatinolytic activities of the HT 1080 cell fraction (C; 13.7 mU of cathepsin L activity) and culture medium (M; 4.33 mU of cathepsin L activity). The activities were estimated in the presence (+) or absence (−) of 0.1 mM leupeptin. The gel and gelatin concentrations were 11.5% and 0.8 mg/mL, respectively. The following molecular markers were used: 116.0 kDa, β-galactosidase; 97.4 kDa, phosphorylase b; 66.0 kDa, BSA; 45.0 kDa, ovalbumin; and 31.0 kDa, carbonic anhydrase. (B) Zymogram of the gelatinolytic activities of the HT 1080 cell fraction (C), lysosomal fraction (L), and culture medium (M), obtained by using the improved gelatin zymography technique with leupeptin.

HT 1080 cell fraction (0.4 μL; 13.7 mU of cathepsin L activity) and culture medium (0.4 μL; 4.33 mU of cathepsin L activity) were subjected to gelatin zymography in the presence or absence of leupeptin. As shown in Fig 1A, very few gelatinolytic active bands were detected in the absence of leupeptin. By contrast, with leupeptin treatment, at least five bands (two 32- and 34-kDa forms with strong activities and three 28.5-, 37-, and 39-kDa forms with faint activities) were detected in the cell fraction; moreover, a 32-kDa form with strong activity and a 41-kDa form with faint activity were detected in the medium. However, no 28.5-kDa band was detected in the medium. These results suggest that leupeptin effectively complexed with the cathepsin L derivatives, including the mature form, and protected them from irreversible denaturation in the electrode alkaline solutions. Moreover, binding of leupeptin during SDS-PAGE enabled visualization of the cathepsin L derivatives, including the 28.5-kDa mature form, as gelatin-degrading activities by gelatin zymography (Fig 1A). As shown in Fig 1B, the lysosomal cathepsin L had a molecular mass of 34 kDa, while the secreted cathepsin L had a molecular mass of 32 kDa. Both forms were detected in the HT 1080 cell fraction as well.

Gelatinolytic activity (with an optimum pH of 4.8) in all forms required thiol reagents and was inhibited by cysteine proteinase inhibitors such as E-64, leupeptin, or N-acetyl-Leu-Leu-methional, but not by ethylenediaminetetraacetic acid, an inhibitor of metalloproteinases, phenylmethylsulphonylfluoride, an inhibitor of serine proteinases, or pepstatin A, an inhibitor of aspartic proteinases [17]. Moreover, we confirmed that these enzymes are all cathepsin L-type proteinases by inhibition analysis performed using cathepsin L specific inhibitors such as CLIK-148 and CLIK-181 [17,20]. Both the 32- and 41-kDa forms in the medium were previously confirmed to represent cathepsin L by immunoblot analysis [17].

### Determination of the carbohydrate species present on 32- and 34-kDa cathepsin L

Both the 32- and 34-kDa forms were detected in the adsorption fraction of the Con A-agarose column (Table 1). In the case of LCA-, RCA120-, and WGA-agarose columns, both forms were detected in the non-adsorption fraction. Among the various lectins, both the 32- and 34-kDa forms bound only Con A, but not LCA, RCA120, or WGA. These results suggest that both forms harbored *N*-linked high-mannose-type oligosaccharides.

**Table 1:** Fractions in which the 32- and 34-kDa cathepsin L forms were detected after running through the lectin columns.

Lectin	Cathepsin L	
	32-kDa form	34-kDa form
**Con A**	adsorption fraction	adsorption fraction
**LCA**	non-adsorption fraction	non-adsorption fraction
**RCA120**	non-adsorption fraction	non-adsorption fraction
**WGA**	non-adsorption fraction	non-adsorption fraction

Oligosaccharide species bound to the 32- and/or 34-kDa cathepsins L were examined using a Lectin-agarose minicolumn Kit I packed with Con A (concanavalin A), LCA (*Lens culinaris* agglutinin), RCA120 (*Ricinus communis* agglutinin 120), and WGA (wheat germ agglutinin), as described in the Materials and Methods. Our improved gelatin zymography method was performed to examine whether the 32- and/or 34-kDa forms were bound to the lectins.

### Examination of the core proteins of 32- and 34-kDa cathepsin L

**Fig 2:**
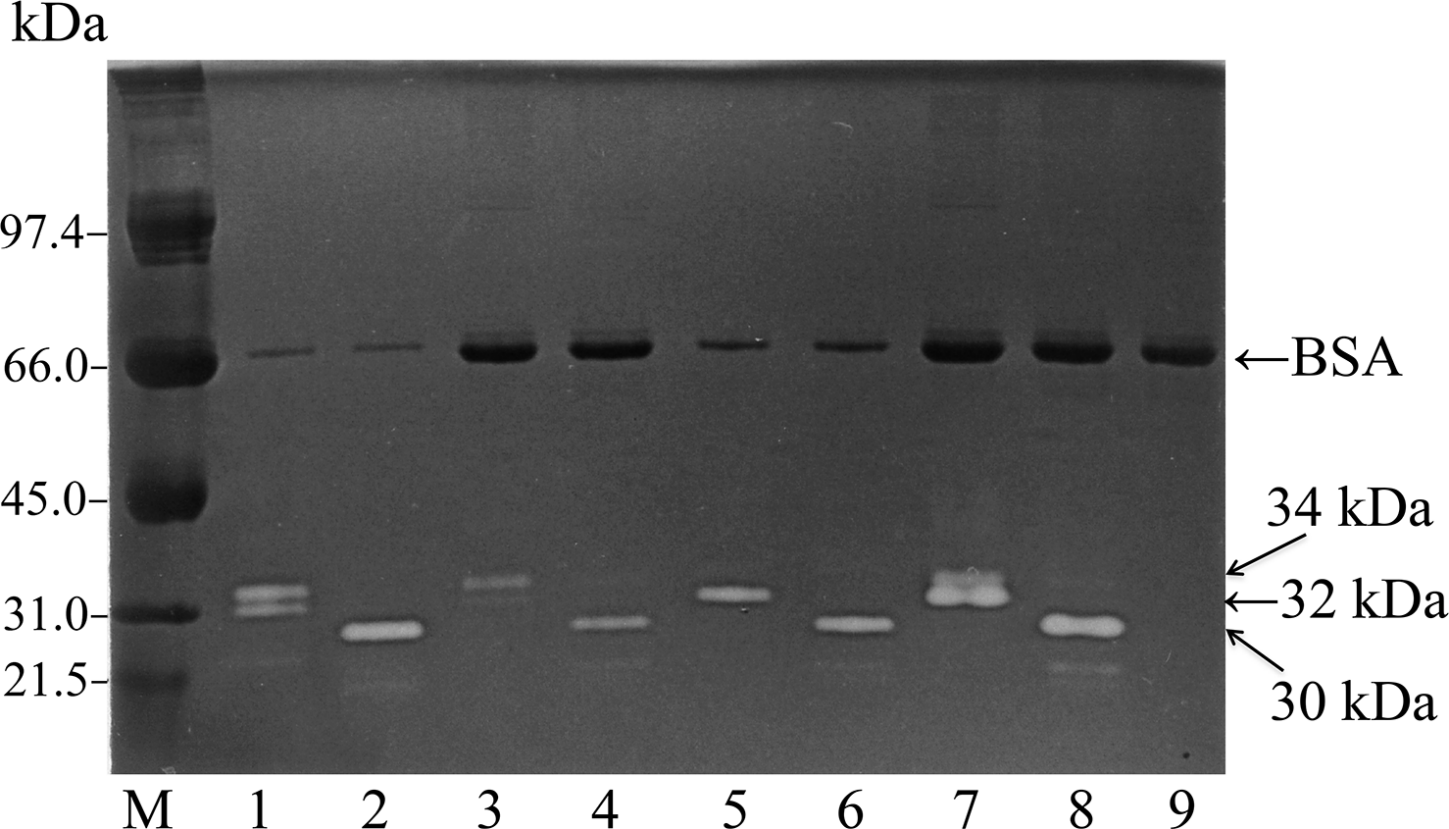
Comparison of the core proteins of 32- and 34-kDa cathepsin L by endoglycosidase H treatment. An aliquot of the cell fraction, lysosomal fraction, or culture medium was mixed with or without 10 mU of End H solution in 0.1 M sodium acetate buffer (pH 5.0) containing 0.1 mM leupeptin, and then incubated for 30 min at 37°C. The improved gelatin zymography technique was used for each incubation mixture. Gelatin zymograms of the cell fraction (lanes 1 and 2), lysosomal fraction (lanes 3 and 4), and culture medium (lanes 5 and 6) with (lanes 2, 4, 6, and 8) or without (lanes 1, 3, 5, and 7) End H treatment are shown. Lanes 7 and 8 show the chromatograms of a mixture of samples from lanes 3 and 5, and from lanes 4 and 6, respectively. Lane 9 shows the zymogram of the incubation buffer alone. The following molecular markers were used: 97.4 kDa, phosphorylase b; 66.0 kDa, BSA; 45.0 kDa, ovalbumin; 31.0 kDa, carbonic anhydrase; and 21.5 kDa, soybean trypsin inhibitor.

End H from *Streptomyces griseus* completely hydrolyzes the GlcNAc—GlcNAc glycoside bond [24–26] and removes high mannose-*N*-linked oligosaccharide units from peptides/proteins [14]. As shown in Fig 2, both 32- and 34-kDa cathepsin L shifted their positions to 30 kDa after End H treatment, suggesting that both forms were End H-sensitive and that the core proteins of both forms were identical. In addition, the core protein alone retained gelatinolytic activity, suggesting that the attached carbohydrate chain was not essential for gelatinolytic activity. Similarly, Smith et al. [27] reported that End H treatment removes a 2-kDa fragment from the 39-kDa mouse procathepsin L; however, the processed form retains complete proteolytic activity.

### Examination of the phosphorylation status of the 32- and 34-kDa cathepsin L forms by treatment with alkaline phosphatase (ALP)

**Fig 3:**
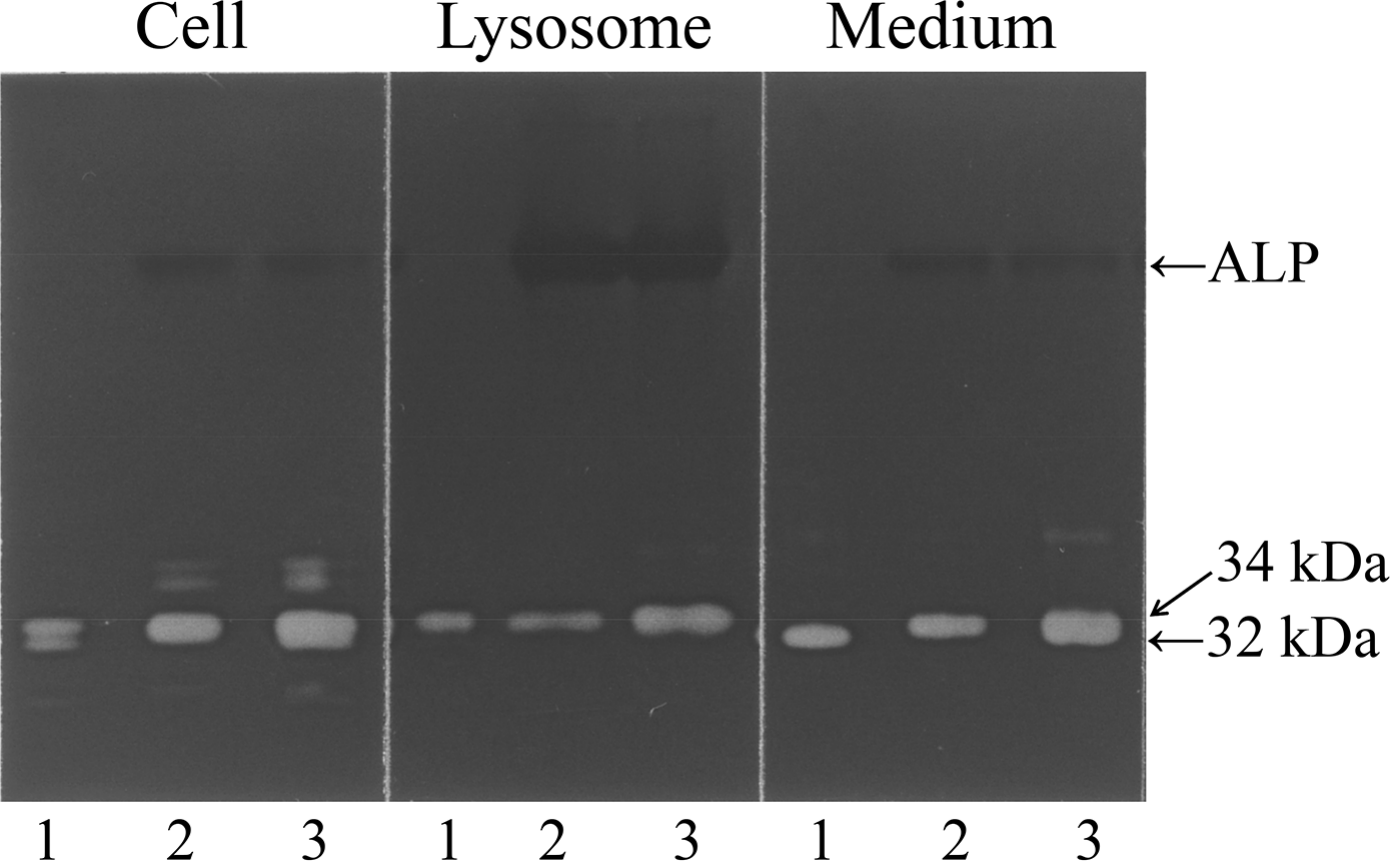
Examination of phosphorylation status of 32- and 34-kDa cathepsin L by treatment with alkaline phosphatase. An aliquot of the cell fraction, lysosomal fraction (34-kDa cathepsin L), or culture medium (32-kDa cathepsin L) was mixed with or without ALP in 0.5 mM Tris-HCl buffer (pH 9.3 at 25°C) containing 0.1 mM leupeptin, 0.1 mM MgCl_2_, 10 μM ZnCl_2_, and 0.1 mM spermidine, and incubated for 30 min at 37°C. Gelatin zymography was performed for each incubation mixture. Gelatin zymograms of the cell, lysosomal, and medium fractions with (lane 2) or without (lane 1) ALP treatment are shown. Lane 3 shows the chromatogram of a mixture of samples from lanes 1 and 2.

As shown in Fig 3, the 32-kDa cathepsin L form, but not the 34-kDa lysosomal form, shifted to the 34-kDa position after ALP treatment, suggesting that the 32-kDa form was phosphorylated, while the 34-kDa form was already dephosphorylated. This discrepancy may be explained as follows: the 32-kDa form might migrate to the anode faster than the dephosphorylated 34-kDa form because of the retention of the negative charge by the phosphate moiety. Acid phosphatase might remove the phosphorus group from the 32-kDa form as soon as it enters the lysosomes, thereby converting it to the 34-kDa form. The phosphorylated status of 32-kDa cathepsin L suggests that the secreted form has never entered lysosomes.

### Mechanism underlying the membrane trafficking of the 32- and 34-kDa cathepsin L forms

**Fig 4:**
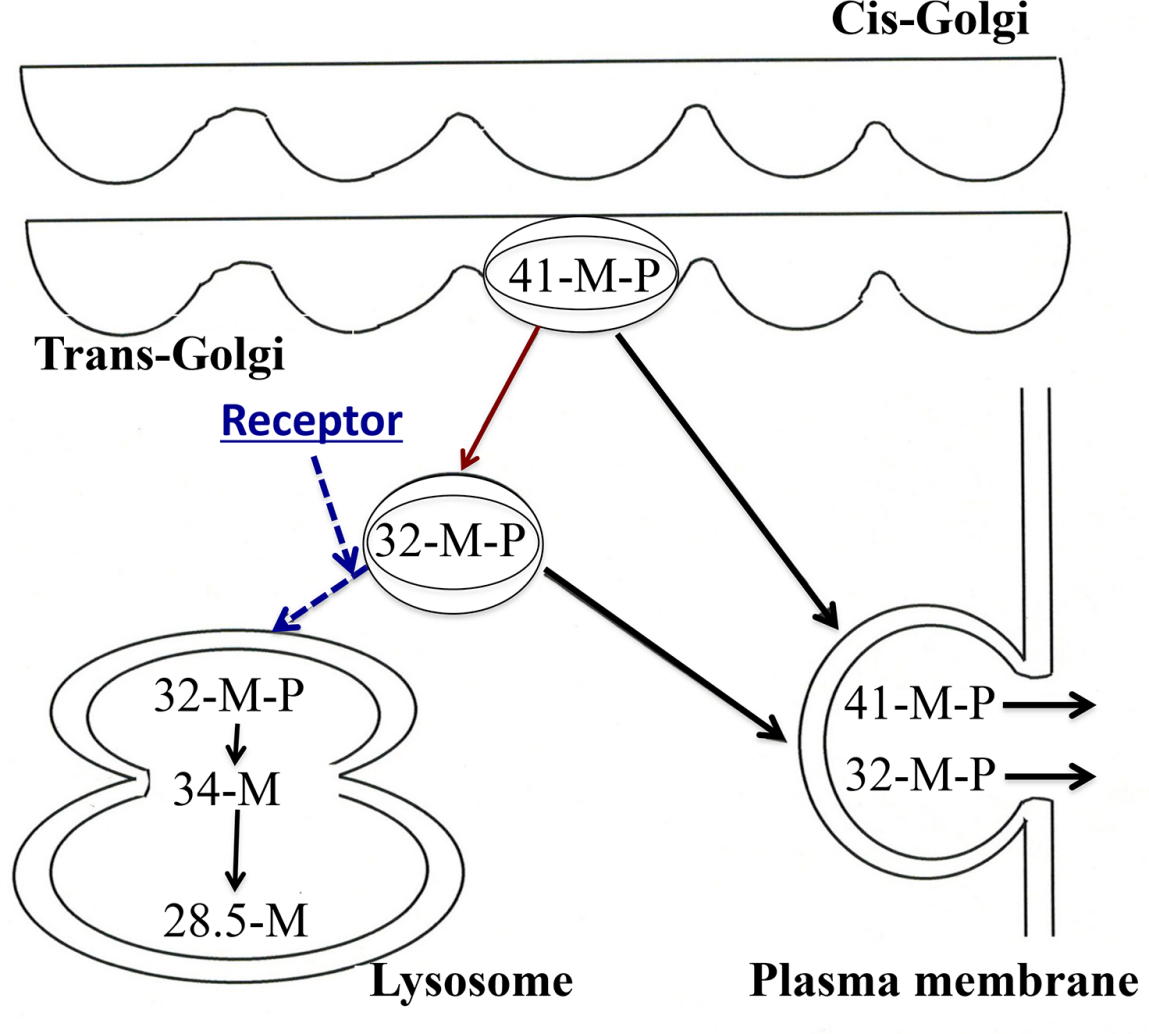
Scheme summarizing the membrane trafficking of active 32-kDa cathepsin L. The 32-kDa cathepsin L form is directly secreted from the TGN after undergoing phosphorylation, and without binding to the M-6-P receptor or entering the lysosomes. 41-M-P, 32-M-P, 34-M, and 28.5-M denote 41-kDa procathepsin L, 32-kDa cathepsin L, 34-kDa cathepsin L, and 28.5-kDa mature cathepsin L, respectively.

A schematic representation of the mechanism underlying the membrane trafficking of the 32- and 34-kDa cathepsin L forms is depicted in Fig 4. The 34-kDa cathepsin L is transported into lysosomes via the M-6-P receptor system. In the acidic conditions of the lysosomes, cathepsin L dissociates from the M-6-P receptor [15], and its phosphate moiety is removed by the lysosomal marker enzyme, acid phosphatase [8]. In contrast, the 32-kDa cathepsin L form does not enter the lysosome and remains phosphorylated. The only difference between the 32-kDa and 34-kDa forms is their phosphorylation status. The 32-kDa form does not harbor a specific signal for secretion.

### Examination of the processing of the 32- and 34-kDa cathepsin L forms

**Fig 5:**
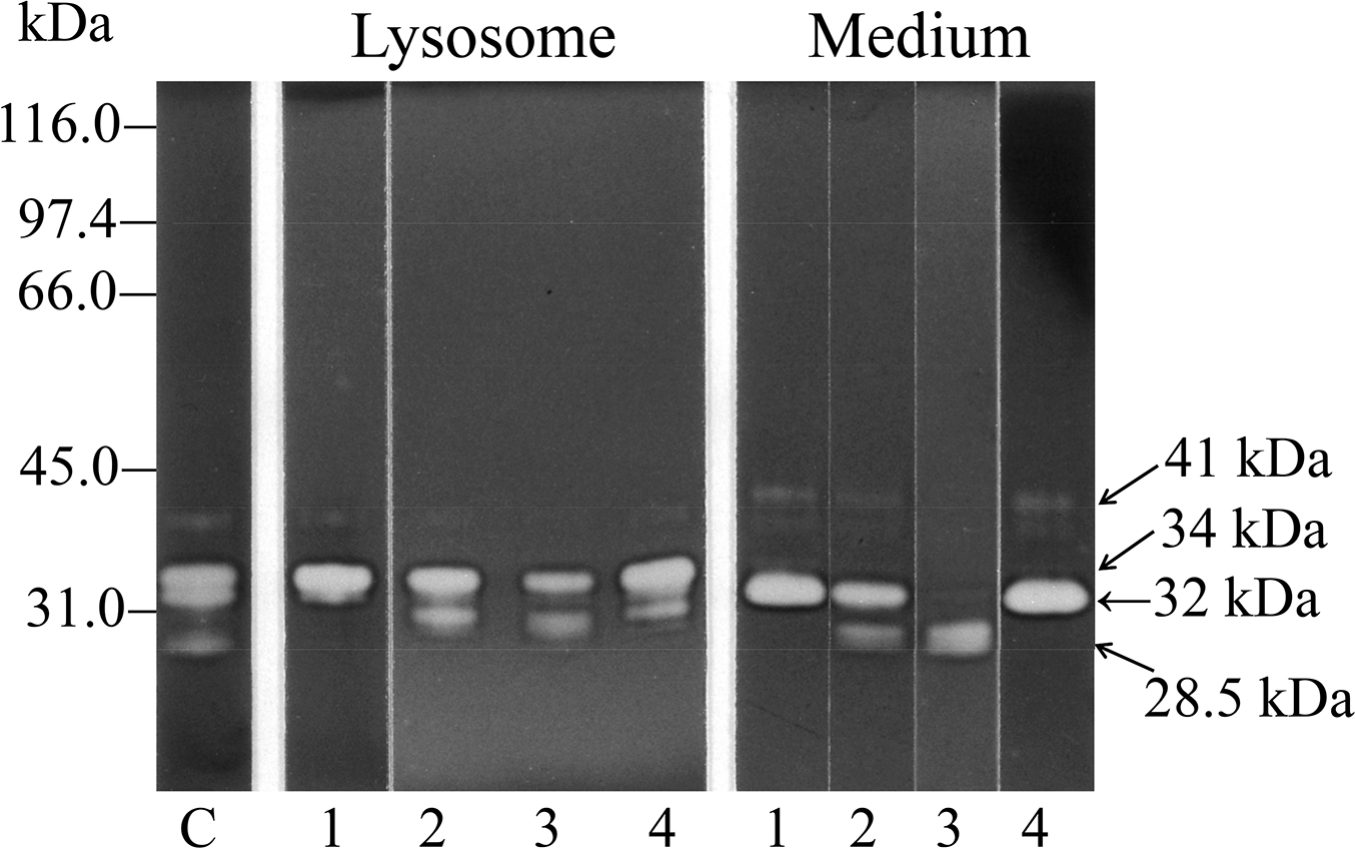
Activation and processing of the 32- and 34-kDa single-chain cathepsins L in acidic solution. The lysosomal fraction (34-kDa cathepsin L) or medium fraction (32-kDa cathepsin L) was incubated in 0.1 M sodium acetate buffer (pH 4.8) for 0 h (lane 1), 2 h (lane 2), or 4 h (lanes 3 and 4) at 37°C in the presence (lane 4) or absence (lanes 1−3) of leupeptin. Lane C shows the zymogram of the cell fraction prior to incubation. After incubation, aliquots of the incubation mixtures and cell fraction (lane C) were analyzed by the improved gelatin zymography technique. The same molecular markers listed in the legend of Fig 1A were used in this experiment as well.

As shown in Fig 5, both the 32- and 34-kDa single-chain cathepsin L forms were processed further to a 28.5-kDa double-chain mature cathepsin L form upon incubation at pH 4.8, suggesting that both were intermediate forms. Leupeptin inhibited this processing at an acidic pH of 4.8. Aspartic proteinases and metalloproteinases participate in the processing, as evidenced by the fact that pepstatin A and 1,10-phenanthrolin partially inhibit the processing of the proform to the mature enzyme [28,29]. Autoactivation of procathepsin L to the mature form occurs at pH 3.0; however, at pH 5.5−6.0, autoactivation occurs only in the presence of negatively charged materials such as dextran sulfate [30]. In the present study, an acidic pH of 4.8 was sufficient to initiate the autoactivation of single-chain cathepsin L intermediates into the mature double-chain form; this process was inhibited by leupeptin. The tumor microenvironment is known to be acidic in nature [31,32]. The secreted 32-kDa intermediate form by itself exhibits high activity [17]. However, in cancer cells, this intermediate form might be rendered more active (similar to the 28.5-kDa mature form) by the acidic conditions in the microenvironment of cancer tissues (Fig 5). However, why the 28.5-kDa mature form is not present in the lysosomes (Fig 1B), despite the acidic conditions, is not clear.

## Discussion

Our results demonstrate that the major active form of cathepsin L in the lysosomes of the HT 1080 cells is 34-kDa cathepsin L, which is glycosylated and dephosphorylated. However, in the culture medium, the major active form is 32-kDa cathepsin L, which is glycosylated and phosphorylated (Figs 1, 2 and 3). The only difference between the two forms is their phosphorylation status. The 32-kDa form does not harbor a specific signal for secretion. Therefore, we hypothesize that a nonsignal constitutive secretory pathway (default pathway) [33] transports the active 32-kDa single-chain cathepsin L directly to the cell surface from the TGN in the HT 1080 cells [34]. This secretion pathway for active cathepsins is distinct from lysosomal exocytosis, and the rate of secretion is faster than that via lysosomal exocytosis [33]. Similarly, Linebaugh et al. [35] reported that viable cells secrete an active 31-kDa single-chain cathepsin B form. This exocytosis of cathepsin B is not related to the secretion of its proenzyme or secretion from mature lysosomes. The authors suggest an alternative pathway for the exocytosis of active cathepsin B.

Newly synthesized lysosomal hydrolases (cathepsin L) are transported to lysosomes via endosomes [36]. The sequential action of two enzymes in the *cis* and TGN adds M-6-P groups to the precursors of lysosomal enzymes. The M-6-P-tagged hydrolases then segregate from all the other types of proteins in the TGN. Transmembrane M-6-P receptors then bind to the AP-1 adaptor complex, which contains clathrin-coated vesicles, on the cytosolic side, and to the M-6-P-modified lysosomal hydrolases on the luminal side [36,37]. The clathrin-coated vesicles bud off from the TGN, shed their coat, and fuse with early endosomes. In the lower pH conditions of the lysosome (endosome), the hydrolases dissociate from the M-6-P receptors [15]. Subsequently, the phosphate moiety is removed from the M-6-P attached to the hydrolases [8]. This explains why dephosphorylated 34-kDa cathepsin L was detected in the lysosomes. The secreted 32-kDa cathepsin L was still phosphorylated, indicating that this protein was secreted directly, without entering the lysosomes.

Kuliawat et al. [38] reported that procathepsin B is efficiently sorted to the lysosomes because it binds to the M-6-P receptor with high affinity. On the other hand, procathepsin L remains in the secretory vesicles because of its low affinity for the M-6-P receptor [38–40]. These findings suggested that cathepsins exit from maturing granules via an M-6-P receptor-dependent mechanism. Similarly, cathepsin L may be secreted into the medium because of its weak affinity for the M-6-P receptor. Moreover, owing to the increased expression of cathepsin B and changes in intracellular trafficking, several human cancers exhibit increased secretion of procathepsin B; additionally, cancer cells secrete active cathepsin B [41]. We previously demonstrated high expression levels of 32-kDa cathepsin L in colon and lung cancer tissue extracts [17]. Although M-6-P receptors are recycled [15,36], the concentration of M-6-P receptor proteins in cancer cells may be too low to deliver the increased number of cathepsin molecules to lysosomes. Moreover, in the case of cancers, the expression level of M-6-P receptor mRNA may be downregulated to ensure increased cathepsin delivery to the extracellular environment; further experiments are required to verify this hypothesis. A candidate drug that enhances the synthesis of M-6-P receptor protein in the cell membrane and/or the Golgi apparatus may be able to reduce the number of extracellular cathepsins by promoting receptor-mediated endocytosis and/or by transporting the cathepsins to the lysosome properly, respectively, and thereby prevent invasion and metastasis.

Inhibition of invasion and metastasis is the method-of-choice for the treatment of cancers. Many cancer cells secrete active cathepsins into the extracellular environment. The secreted cathepsins then degrade the extracellular matrix, and enable invasion and metastasis in the acidic microenvironment. Therefore, studies investigating the mechanisms underlying the secretion of active cathepsins by cancer cells may lead to the development of novel cancer therapies.

In conclusion, our results suggest that the active 32-kDa cathepsin L form detected in the culture medium is secreted directly from the HT 1080 cells and not via lysosomal exocytosis or extracellular processing of procathepsin L.
